# Treatment of Secondary Chorea: A Review of the Current Literature

**DOI:** 10.5334/tohm.351

**Published:** 2020-07-16

**Authors:** Erin Feinstein, Ruth Walker

**Affiliations:** 1 Rutgers, US; 2 Icahn School of Medicine at Mount Sinai, US

**Keywords:** chorea, VMAT2 inhibitor, tardive dyskinesia, levodopa-induced dyskinesia, amphetamine, chorea gravidarum, autoimmune, brain iron accumulation disorders

## Abstract

**Background:**

Chorea consists of involuntary movements affecting the limbs, trunk, neck or face, that can move from one body part to another. Chorea is conceptualized as being “primary” when it is attributed to Huntington’s disease (HD) or other genetic etiologies, or “secondary” when it is related to infectious, pharmacologic, metabolic, autoimmune disorders, or paraneoplastic syndromes. The mainstay of the secondary chorea management is treating the underlying causative disorder; here we review the literature regarding secondary chorea. We also discuss the management of several non-HD genetic diseases in which chorea can be a feature, where metabolic targets may be amenable to intervention and chorea reduction.

**Methods:**

A PubMed literature search was performed for articles relating to chorea and its medical and surgical management. We reviewed the articles and cross-references of pertinent articles to assess the current clinical practice, expert opinion, and evidence-based medicine to synthesize recommendations for the management of secondary chorea.

**Results:**

There are very few double-blind randomized controlled trials assessing chorea treatments regardless of etiology. Most recommendations are based on small open-label studies, case reports, and expert opinion.

**Discussion:**

Treatment of secondary chorea is currently based on expert opinion, clinical experience, and small case studies, with limited evidence-based medical data. When chorea is secondary to an underlying infection, medication, metabolic abnormality, autoimmune process, or paraneoplastic illness, the movements typically resolve following treatment of the underlying disease. Tardive dyskinesia is most rigorously studied secondary chorea with the best evidence-based medicine treatment guidelines recommending the use of pre-synaptic dopamine-depleting agents. Even though there is an insufficient pool of EBM, small clinical trials, case reports, and expert opinion are valuable for guiding treatment and improving the quality of life for patients with chorea.

**Highlights:**

There is a dearth of well-controlled studies regarding the treatment of chorea. Expert opinion and clinical experiences are fundamental in guiding chorea management and determining successful treatment. In general, secondary chorea improves with treating the underlying medical abnormality; treatments include antibiotics, antivirals, immunosuppression, dopamine depleting agents, chelation, and supportive care.

## Introduction

Chorea is a hyperkinetic movement disorder consisting of rapid involuntary movements that flow from part of the body to another. The mechanism generating this movement disorder is hypothesized to result from an imbalance of neurotransmission in the direct and indirect pathways of the basal ganglia (BG). Dopamine (DA) has a net excitatory effect on the thalamus via the direct and indirect pathways, causing increased cortical signaling. DA stimulates the direct pathway through the activation of excitatory DA D1 receptors on GABA-ergic neurons in the caudate-putamen which project to the globus pallidus pars interna (GPi) facilitating movement. DA inhibits movement via the indirect pathway with stimulation of inhibitory DA D2 receptors located on GABA-ergic neurons of the striatum that project to the external segment of the globus pallidus (GPe), then to the subthalamic nucleus (STN). According to the model, decreased activity of the indirect pathway and/or increased activity of the direct pathway results in a decrease of the overall inhibitory effect of the BG, causing increased thalamo-cortical output that can present as chorea.

Tardive dyskinesia (TD) and levodopa-induced dysknesia (LID) may both result from dysregulation of the direct and indirect pathways. TD is potentially caused by a heightened sensitivity of the D2 receptor from chronic DA blockade, decreasing indirect pathway activity and allowing for the breakthrough of abnormal movements [1]. Chronic DA replacement may increase sensitivity of the D1 receptor resulting in hyperactivity of the direct pathway allowing for LID. Because of this pathophysiology and the frequent choreiform phenotype of TD and LID, they will be considered forms of chorea in the context of this paper. Other models of secondary chorea are discussed in their subsequent sections.

There are many medical conditions that can present with chorea, including infections/post-infectious syndromes, pharmacological agents – both prescribed and used recreationally, metabolic disorders, pregnancy, autoimmune disorders, paraneoplastic syndromes, and genetic abnormalities [2]. Primary chorea treatment is typically targeted at reducing chorea; secondary chorea management consists of treating the underlying medical condition that is causing chorea.

At present, treatment of chorea is deduced from clinical experience, anecdotal evidence, expert opinion, small trials, and case reports, with few randomize control trials (RTCs). Challenges to conducting chorea research include the rarity of chorea and chorea-associated syndromes, the variety of neurodegenerative phenotypes with variable progression rates, and the inherent self-limiting pattern of most chorea. Neurodegenerative diseases with secondary chorea are especially challenging to study, most participants are selected in the early stages of their disease process and do not represent the clinical spectrum. These studies produce treatment recommendations that are not applicable as the disease progresses. Such discrepancies have led to a divide between clinical treatments and evidence-based medicine (EBM) recommendations. Even though there is an insufficient pool of EBM, small clinical trials, case reports, and expert opinion are valuable for guiding treatment and improving the quality of life for patients with chorea.

This paper focuses on the treatment and management of secondary chorea as recommended from EBM, expert opinion, case studies, RTCs, and small open-label clinical trials, with a review of each treatment.

## Methods

We conducted separate PubMed database searches for English language articles, utilizing the terms “chorea,” in combination with disease-related terms “infection,” “Sydenham,” “human immunodeficiency virus,” “tardive dyskinesia,” “estrogen,” “amphetamine,” “cocaine,” “B12,” “chorea gravidarum,” “autoimmune,” “paraneoplastic,” “neurodegeneration with brain iron accumulation syndromes [NBIA],” “Wilson’s disease,” in combination with treatment-related terms “treatment,” “penicillin,” “antibiotic,” “corticosteroid,” “IVIg,” “plasmapheresis,” “valproic acid,” “anticonvulsants,” “antipsychotic,” “tetrabenazine,” “deuterated tetrabenazine,” “valbenazine,” “chelating agent,” “chelation.”

This search identified 766 articles. All articles were screened by reviewing the title and abstract to determine if they fell within the scope of this paper. Articles were excluded if they were not in English, if no abstract was available, or if they included medications not available or withdrawn from the US market. Articles were excluded due to lack of relevance (not related to chorea or chorea treatment, outdated information, or redundancy). From the total 766 articles identified, 97 were selected for further review, being directly related to treatment. The search included human and nonhuman subjects. Articles were published between 1971 and November 2019. The search was first performed in December 2015 and updated to be current until February 1^st^, 2020.

## Results

When chorea presents in the setting of an underlying disease, disease-specific therapy is often the most effective treatment. See Table 1 for a summary of causes of secondary chorea with empiric and symptomatic treatment recommendations.

**Table 1:** Summary of causes of secondary chorea and empiric and symptomatic treatment recommendations. Defined abbreviations: ANNA-1, 2: anti-neuronal nuclear antibodies type 1 and 2, aCP: aceruloplasminemia, ASA: aspirin, ASP: antiphospholipid syndrome, CBZ: carbamazepine, CASPER2: contactin-associated protein-like 2, CRMP5: collapsin response mediator protein 5, DA: dopamine, DBS: deep brain stimulation, deuTBZ: deuterated TBZ, D2: dopamine 2 receptor, GAD65: glutamic acid decarboxylase, GLT1: glucose transport 1, GPi: globus pallidus interna, HAART: highly-active antiretroviral therapy, HIV: human immunodeficiency virus, HSV-6: herpes virus-6, IVIg: intravenous immunoglobulin, LID: levodopa induced dyskinesia, NBIA: neurodegeneration with brain iron accumulation disorders, Nf: neuroferritinopathy, PKD: kinesigenic dyskinesia, PnKD: non-kinesigenic dyskinesia, SLE: Systemic lupus erythematous, STN: subthalamic nucleus, TD: tardive dyskinesia, TBZ: tetrabenazine, WNV: West Nile virus, VPA: valproic Acid, VZV: varicella zoster virus.

Cause of secondary chorea	Etiologic treatment	Symptomatic treatment

**1) Infectious**		

Sydenham chorea	Acute phase: penicillin 500 mg BID for 10 days or 1 IM dose Chronic phase: penicillin G 1.2 million daily for 21 days	VPA 5–20 mg/kg/day CBZ 15–20 mg/kg/day IVIg 1 mg/kg/day
VZV		ASA 81 mg/day VPA 5 mg/kg/day
HSV-6	Foscarnet 40 mg/day for 2 weeks	IVIg 1 g/kg/day for 5 days
Tick encephalitis		Haloperidol Dexamethasone Pentoxifylline Nitrazepam Midazolam
Influenza A, parvovirus B19 encephalitis, WNV		Supportive care
Syphilis	Benzathine penicillin 2.4 million units IV Penicillin G 2.4 million units IM for 14 days	
HIV	HAART	
HIV with toxoplasmosis	HAART Pyrimethamine sulfadiazine	
**2) Drug-induced**		

Cocaine		Drug cessation Supportive care
Methamphetamine		Drug cessation Supportive care
TD	TBZ 12–100 mg/day deuTBZ 24 or 36 mg/day VBZ 40 or 80 mg/day	Levetiracetam 500 mg- 300 mg daily
LID	DBS of GPi or STN Amantadine >200 mg/day	Medication cessation Medication reduction
**3) Metabolic and Pregnancy-related**		

Diabetic non-ketotic hyperglycemia	Insulin	Supportive care
Hyperthyroidism	Methimazole 3mg/day	Beta-blocker (propranolol 20–60 mg/day, metoprolol 25 mg/day) DA D2 blocking agent (chlorpromazine)
Chorea gravidarum	Birth	
Estrogen supplementation		Cessation of estrogen
Cobalamin deficiency	Acute: cyanocobalamin 1000 mcg/day for 10 days Chronic: cyanocobalamin 1000 mg/daily for 6 months	
**4) Autoimmune and paraneoplastic**		

APD	Anti-platelets Anticoagulation	IVIg Plasmapheresis Immunosuppressive agents (mycophenolate, methotrexate)
SLE	Anticoagulation	Methylprednisolone 500 mg/daily for 5 days IVIg
LGI1		Immunosuppression (methylprednisolone 1000 mg/day)
Celiac disease	Gluten-free diet	
Paraneoplastic and non-paraneoplastic diseases (ANNA-1,2, anti-CASPR2, anti-CRMP5, anti-LGI1, anti-NMDA, anti-Hu, anti-Ri, anti-Yo, anti-GAD65)	Oncologic targeted treatment	Immunosuppression Corticosteroids Cyclophosphamide IVIg
**5) Genetic non-HD**		

GLUT1 deficiency	Ketogenic diet	
PKD		Phenytoin CBZ Benzodiazepines VPA Gabapentin Lamotrigine Levetiracetam Oxcarbazepine
PnKD	Trigger avoidance	Benzodiazepines
aCP	Chelating agents (deferasirox 1000 mg/day or desferrioxamine 1000 mg/day for 5 day) Deferiprone 500 mg/day with fresh frozen plasma Ceruloplasmin	
NBIA	Chelating agents (deferiprone 15 mg/kg BID	
Nf		DA modulating agents (sulpiride 400 mg/day, TBZ up to 125 mg/day)
Wilson’s disease	Chelating agents (Trientine, D-Penicillamine, tetrathiomolybdate) Zinc	

### Infectious causes of chorea

Chorea that appears following an infectious process is likely due to a multifactorial mechanism, such as a combination of neurotropic viral properties, as-yet-unidentified cross-reacting antibodies affecting the neurons of the BG, and infectious susceptibility of deep brain tissue [3,4]. Choreiform movements may present in the subacute time frame and improve within days to months after initiating treatment, depending on the infectious cause.

The most common form of childhood chorea is Sydenham’s chorea (SC), in which patients present with chorea during the recovery phase of a group A streptococcal infection. Chorea is thought to be due to a cross-reactivity of disease-specific antibodies in SC that recognize lysogangliosides on neuronal cell surfaces in the striatum, resulting in dysregulated BG activity [5,6]. The first line of treatment for SC is penicillin 500 mg twice daily for 10 days or a single intramuscular (IM) dose followed by chronic penicillin G 1.2 million units every 21 days depending on the severity of cardiac damage, as recommended by the World Health Organization [7]. In cases where chorea does not resolve or is severe, symptomatic treatment of DA blocking agents, antiepileptic drugs (AEDs), or immunomodulatory therapies such as corticosteroids, intravenous immunoglobulin (IVIg), or plasma exchange are indicated. In a review of 65 SC cases treated with open-label haloperidol (0.05 mg/kg/day two to three times a day) there was improved recovery time; however, side effects include parkinsonism and TD, making haloperidol not the treatment of choice for the pediatric population [7,8]. Case reports and small case studies using AEDs such as valproic acid (VPA) 15-20 mg/kg/day and carbamazepine (CBZ) 15–20 mg/kg/day twice a day can effectively improve chorea within days [9,10]. Long term use of VPA in the pediatric population is not recommended as it may result in premature growth plate ossification, increased risk of developing polycystic ovarian syndrome, and the risk of birth defects in pregnancy. In a randomized-entry controlled trial of 18 people with SC treated with IVIg 1 g/kg/day for two days or alternate day plasma exchange for five to six days improved faster and more robustly than those treated with prednisone 1 mg/kg/day for 10 days [11].

Chorea has been reported in children following varicella zoster virus (VZV) and herpes simplex (HSV) encephalopathy. One pediatric case of VZV with chorea improved within one week of aspirin 5 mg/kg/day and VPA [12]. A case of HSV-6 encephalitis with chorea improved with 5 doses of IVIg and 2 weeks of foscarnet i.v [13].

Chorea rarely presents due to infectious etiologies in the adult population; in these cases chorea improves with appropriate antibiotic or antiviral therapy. There is one case of tick-born encephalitis presenting with chorea that improved with haloperidol, dexamethasone, pentoxifylline, nitrazepam, and midazolam [14]. Cases of influenza A, parvovirus B19 encephalitis, and West Nile virus encephalitis presenting with chorea that slowly improved with supportive care over several weeks [15,16,17]. When syphilis was the cause, symptoms responded to standard treatment with benzathine penicillin 2.4 million units IM followed by penicillin G 2.4 million units IM for 14 days [18,19]. In a number of cases, patients were co-infected with syphilis and HIV requiring both antibiotic and antiviral treatments [19,20]. Chorea as a presentation of HIV alone may improve with antiretroviral therapies (zodovudine 1,200 mg/day, didanosine 600 mg/day, ritronavir 10 mg/day [21].

When chorea is seen in the setting of opportunistic infections with HIV, the movement disorder improves with antiviral and antibiotic treatment of the underlying infectious lesion. Most toxoplasmosis-HIV infections presenting with chorea were reported in the late 1990s, before the widespread access to long-term highly-active antiretroviral therapy (HAART), which was released in 1997. Patients with HIV/AIDS toxoplasmosis with chorea treated with pyrimethamine, sulfadiazine, and HAART showed improvement or resolution of chorea in days to weeks of initiating treatment, and patients who were not started on HAART did not show such improvement [22,23,24,25]. In a recent case of HIV-related histoplasmosis infection with chorea, the movements resolved within days of starting amphotericin B and itraconazole while continuing HAART [26]. Since the development of advanced HAART, the number of cases of HIV-related chorea and HIV-opportunistic infection-related chorea has significantly decreased, highlighting the importance of advancement in HIV treatment. These case reports emphasize the importance of correctly identifying and treating the underlying infectious pathology in the management of secondary chorea.

### Drug-induced chorea

Multiple recreationally-used substances and medications can potentially cause chorea by increasing DA levels or by dysregulation of the direct and indirect pathways. Typically, medication-induced chorea improves with cessation of the offending agent and the passage of time.

Cocaine is the most commonly used illicit drug that presents with chorea. Cocaine toxicity can present with a well-documented chorea known as “crack dancing” due to blockade of presynaptic DA uptake, and hence increased DA levels in the BG. Cocaine-induced chorea typically resolves with supportive care within three to 12 days [27,28].

Amphetamine use may also present with chorea, due to accidental ingestion in the pediatric population, over medication in patients with attention deficit disorder (ADHD), and recreational use in the adult population. Accidental ingestion of amphetamine salts caused chorea in an 8-month-old boy who had symptomatic resolution within in 72 hours of admission to pediatric ICU and supportive care [29]. There are two cases of ADHD patients who developed chorea after increased doses of amphetamine salts. A 10-year-old boy with ADHD accidentally received an additional dose of lisdexamfetamine resulting in chorea that improved with pediatric ICU care and administration of haloperidol 48 hours after ingestion [29]. And a 22-year-old man with ADHD who was stable on mixed amphetamine salts for 14 years required constant dose increases during his college years. The increase to 45 mg TID produced choreiform movements, anxiety, and pressured speech, all of which resolved within 48 hours of medication termination [30]. Six cases of adult recreational amphetamine use causing limb, neck, and facial chorea resolved with supportive care within 24 hours [30,31,32,33]. Regardless of the causative agent, severe drug-induced chorea may require haloperidol or lorazepam to suppress the movements, and possibly intensive care unit (ICU) management for autonomic instability [34].

Long term and short-term use of DA blocking agents can cause TD. Unlike most other drug-induced chorea, TD does not typically resolve with cessation of the offending agent and can be challenging to treat, requiring sophisticated medical management and possibly surgical intervention [1]. Avoidance of DA-blocking agents prevents the development of TD; this is obviously not an option for psychiatric patients for whom such medications are essential for mental health. TD has been studied in more double-blind placebo controlled RTCs, employing consistent use of formal chorea scoring, than any other secondary chorea.

TD improves with depletion of presynaptic DA and hence decreased DA release, which can be achieved with the use of vesicular monoamine transporter 2 (VMAT2) inhibitors, such as tetrabenazine (TBZ), deuterated TBZ (deuTBZ), and valbenazine (VBZ). In a meta-analysis of 10 retrospective trials of TBZ in which 1,142 TD patients were treated with doses ranging from 12–100 mg/day, 71% had marked, excellent, or complete improvement of chorea with side effects of depression and increased suicidal thoughts [35]. DeuTBZ is nearly chemically identical to TBZ with the replacement of 2 hydrogen atoms with deuterium, which extends the half-life, allowing for less frequent dosing, more stable serum levels, and fewer side effects. The ARM-TD trial provided class I EBM that deuTBZ can significantly improve abnormal involuntary movement scale (AIMS) scores for TD patients at a mean dose of 40 mg/day, with few reports of anxiety and depression and no reports of suicidal ideation (SI) [36]. The AIM-TD trial was a double-blind RTC which demonstrated that deuTBZ at doses of 24 mg/day and 36 mg/day were effective at reducing AIMS scores with a few reports of psychiatric side effects [37]. Psychiatric side effects included anxiety (4% of treatment group, 2% of placebo group), anxiety disorder (<1% of treatment group), depressed mood (<1% of treatment group), depression (5% of treatment group), irritability (2% of treatment group), and suicidal idealizations (3% of treatment group). Although effective treatments for TD, TBZ and deuTBZ have the potential to worsen psychiatric symptoms of depression and suicidality, thus patients should be monitored closely.

VBZ is a highly selective VMAT2 inhibitor, resulting in fewer side effects than TBZ with a similar reduction in TD symptoms. In the 6 week double-blind RCT (KINECT-3), 234 patients with stable schizophrenia, schizoaffective disorder, or mood disorder with at least 3 months of TD symptoms were treated with either 40 mg or 80 mg VBZ significantly reduced abnormal involuntary movement scale (AIMS) scores [38].

AEDs can also potentially improve TD. In a double-blind RCT of TD patients treated with levetiracetam 500–300 mg/day had significantly reduced AIMS scores with minimal side effects of gastrointestinal upset [39].

Long term use of levodopa for treatment of Parkinson’s Disease (PD) can cause LID. Similar to other drug-induced chorea, LID improves with decreased levodopa dosage or medication cessation. Multiple small RTCs have shown that amantadine can decrease peak LID in PD patients for at least a short duration [40,41,42]. Two large RTCs assessed the long term anti-dyskinetic benefits of amantadine [43,44]. Wolf et al studied 32 PD patients who were stable on an average dose of amantadine 298 mg/day for at least 12 months [43]. Then, in a double blind fashion the participants were switched to receive either their current dose of amantadine or a placebo for 3 weeks. At the end of trial, patients in the placebo group had significantly increased unified PD rating scale (UPDRS) IV items 32 + 33 compared to baseline. Patients who remained on amantadine did not have a significant change from baseline. In the follow-up AMANDYSK trial 57 PD patients stable on amantadine >200 mg/day for 6 months were then switched to either continuation of amantadine or placebo in randomized double-blind groups for 3 months [44]. Placebo-treated patients had significant increased UPDRS IV items 32 + 33 compared to patients who remained on amantadine at the end of the trial. Both of these large scale studies indicate that long term use of amantadine reduces LID’s.

Deep brain stimulation (DBS) is a surgical intervention consisting of electrodes implanted into either the STN or GPi of PD patients with the goal of improved motor function and reduced LID [45]. The exact pathophysiology of DBS is not fully understood, and reduction of LID may be due to stimulation of inhibitory afferents to the GPe or GPi resulting in improved regulation of the direct and indirect pathway [46,47]. Additionally, DBS is used to control PD motor symptoms allowing for an overall reduction of L-dopa dosing [48]. Reduction of L-dopa results in reduced LID by minimizing the causative agent [46].

In conclusion, drug-induced chorea generally responds to cessation or reduced use of the causative agent. Full avoidance is the treatment of choice for cocaine and reduced use or abstinence is also recommended for amphetamine-based drugs. VMAT2 inhibitors are specific treatment options for patients with TD. Amantadine and DBS are LID-specific treatments for PD patients.

### Metabolic and pregnancy-related chorea

Metabolic abnormalities such as hyperglycemia, hyperthyroidism, vitamin B12 deficiency, pregnancy, and others, have been associated with chorea.

Diabetic non-ketotic hyperglycemia is a well-recognized cause of chorea, which is typically asymmetric and associated with typical T1 weighted MRI abnormalities in the contralateral striatum. The underlying pathophysiology generating chorea is not understood. Possible mechanisms include putaminal vascular changes, inhibition of GABA transmission in the striatum, decreased striatal blood flow and metabolism, and/or severe energy depletion and neuronal dysfunction as seen with increased lactic acid, acetatae, and lipids and decreased N-acetylaspartate and creatinine on as seen on MR spectroscopy [49,50,51]. A possible combination of these pathological processes leads to dysfunction of the striatum with hypofunction of the indirect pathway resulting in hemichorea. Typically, the symptoms resolve as the metabolic abnormalities are normalized, although this can take weeks, and in rare cases the movement disorder is permanent.

The first reported case of hyperthyroidism causing chorea was by Sir William Gowers in 1888. In recent case reports, two young women with hyperthyroidism presented with chorea; both were found to have TSH <0.01 IU/mL and free T4 <6 mg/dL. Their symptoms resolved over a few weeks with the use of either metoprolol or chlorpromazine, in addition to methimazole [52,53]. In another case report a 78-year-old woman developed chorea while on thyroxine 175 μg/day; free T4: 41.1 pmol/l and TSH: 0.25 mU/l. With cessation of thyroxine and initiation of propranolol 20 mg daily she became euthyroid after 1 week, although the chorea persisted for 3 months [54]. In general, chorea is rarely seen with hyperthyroidism, and can be managed with the initiation of methimazole 30 mg/day and a beta-blocker (metoprolol 25 mg/day, propranolol 60 mg/day), or D2 blocking agents (chlorpromazine 0.5 to 0.1 mg/day) [52,53,55]. This time disparity between cessation of hormonal treatment and resolution of chorea indicates that some patients continue to have increased BG activity, possibly due to long term hypersensitivity after hormonal exposure. These cases emphasize the rarity of iatrogenic hormonal therapy-induced chorea and the need for medication cessation and monitoring.

Hormonal medications may increase BG sensitivity to DA, or reduce DA turnover, resulting in chorea, for example, in the setting of *chorea gravidarum* [*35*,*36*]. *Chorea gravidarum* occurs during pregnancy, likely due to increased BG sensitivity to DA due to high estrogen setting [56,57]. *Chorea gravidarum*, is more likely to occur in woman who had SC in childhood, and resolves shortly after birth when estrogen levels decrease. [57,58]. Six cases of chorea were reported related to the use of oral contraceptive estrogen pills, which improved within weeks to months of hormone cessation [59,60]. The pathophysiology of *chorea gravidarum* is likely similar, if not identical to that of chorea secondary to estrogen supplementation; both conditions resolve as estrogen levels normalize.

There are 4 reported cases of severe cobalamin deficiency resulting in chorea. In a recent case an 80-year-old woman was found to have chorea and with an undetectable level of vitamin B12 (<83 pg/mL). She was treated with intramuscular cyanocobalamin 1000 mcg daily for 10 days followed by 1000 mcg weekly for 6 months in conjunction with folic acid 5 mg daily. Her chorea improved slightly after 7 days and continued to improve by day 15 [61]. The pathophysiology of chorea secondary to vitamin B12 is not fully understood and may be due to elevated neurotoxic levels of methylmalonic acid, methyl-tetra-hydrofolate, and homocystine resulting in dysregulation of BG function that resolves with appropriate supplementation.

### Autoimmune and paraneoplastic associated chorea

Many autoimmune diseases can present with chorea in adults; these include antiphospholipid syndrome (APS), systemic lupus erythematosus (SLE), Sjogren’s syndrome (SS), rheumatoid arthritis (RA), autoimmune thrombocytopenic purpura (aTP), and Hashimoto thyroiditis (HT) also known as chronic lymphocytic thyroiditis. Additionally, paraneoplastic syndromes can present with chorea. This secondary chorea responds to treatment of the underlying disorder as appropriate, with antiplatelet agents, anticoagulation, immunosuppression, intravenous immunoglobulin, corticosteroids, or plasmapheresis [62,63].

APS is an autoimmune disorder with recurrent venous or arterial thrombosis, thrombocytopenia, and/or recurrent spontaneous abortions in the setting of a positive blood test for antiphospholipid antibodies. It is considered primary if it occurs in isolation and secondary if present in conjunction with SLE or other autoimmune diseases. As such, the pathophysiology of APS related-chorea likely overlaps that of SLE-induced chorea. It has been proposed that anti-phospholipid antibodies bind to intracranial endothelium causing inflammation and increased permeability of the blood brain barrier; hence allowing for direct anti-phospholipid antibody binding to BG neurons [64]. Approximately 1.3% of people with APS develop chorea, and respond to immunosuppression combined with antiplatelet agents, anticoagulation, immunoglobulins, and/or plasmapheresis [63,64,65]. Given this response to immunosuppression, there is likely an element of inflammation and immunologic cross-reactivity between anti-phospholipid antibodies and the BG causing chorea. The pro-thrombotic nature of anti-phospholipid antibodies is likely to contribute to the pathophysiology given the response of chorea to antiplatelet agents and anticoagulation.

There are case reports of APS patients requiring more aggressive immunosuppression than corticosteroids, such as IVIg, mycophenolate, or methotrexate. In one case report a woman with APS had full recovery of chorea after 2 weeks of methotrexate 20 mg/day [66]. There are two case reports of pediatric APS with some resolution of chorea after treatment with 2,000 mg/day mycophenolate mofetil [67]. Similarly, A 33-year-old woman with SLE required methylprednisolone 500 mg iv daily, low-molecular-weight heparin (LMWH) 0.6 mL subcutaneously daily, and IVIg (20 g/day for 5 days) for chorea improvement [68].

Leucine-rich glioma-inactivated 1 (LGI1) proteins are involved in tumor suppression and epilepsy. LGI1 is a target for autoantibodies which bind to voltage-gated potassium channel complexes throughout the brain, LGI1 antibodies are classically associated with limbic encephalitis and faciobrachial dystonic seizures. In one case report, a patient with LGI1 antibody syndrome with chorea responded to a 5-day course of 1000 mg methylprednisolone daily followed by a short taper [69]. This rapid and robust response to corticosteroids is thought to suggests that immunosuppression prevented these antibodies from binding to voltage-gated potassium channels in the BG [69].

Celiac disease is an autoimmune disorder that can present with chorea, in addition to other neurologic symptoms including ataxia, neuropathy, dementia, and seizures, and may improve with a gluten-restricted diet [70,71]. There are case reports of patients with anti-gliadin antibody-confirmed celiac disease and chorea who had improvement in chorea and decreased anti-gliadin antibodies with strict gluten free diet compliance, suggesting cross-reactivity between anti-gliadin antibodies and BG neuronal targets [72].

HT is an autoimmune disease caused by antibodies to thyroid peroxidase, thyroglobulin, and/or thyroid stimulating hormone receptors causing an antibody-dependent cellularly mediated cytotoxicity of the thyroid. Encephalopathy and myoclonus are common neurologic presentations of HT, and there are cases of dystonia, tremor, ataxia, gait disorder, and dysarthria [73]. There are two reported cases of HT presenting with chorea, one without encephalopathy and one with encephalopathy. Both cases improved with immunosuppression and levothyroxin. The first case was a 77-year-old woman with sudden onset of hallucinations, gait disorder, and chorea of the limbs and head [74]. She had elevated anti-thyroglobulin titers and clinically improved with-in days of initiating prednisone 60 mg/day and levothyroxin 75 mcg/day. Her anti-thyroglobulin titers remained high; prednisone was increased to 80 mg/day and within 3 weeks of symptom onset she had returned to baseline with normalization of titers. The second case was a 34-year-old woman with sudden onset of choreoathetosis of the left upper extremity, dysarthria, and abnormal gait [73]. Within 2 years her dysarthria worsened and she progressed to generalized chorea. She had further disease progression over 4 years and was found to have high anti-TPO and anti-thyroglobulin titers. She clinically improved with iv methylprednisolone and IVIg. These cases demonstrate possible cross-reactivity between anti-thyroglobulin and BG neurons.

In a 2013 review of 36 cases of adult-onset autoimmune chorea at the Mayo Clinic (Rochester, MN) 14 patients had a paraneoplastic etiology. These were defined as the onset of chorea within 2 years of a diagnosis of active cancer, or a positive serum or CSF autoantibody with high predictive value for cancer [such as anti-neuronal nuclear antibodies type 1 and 2 (ANNA-1,2), anti-CASPR2, anti-CRMP5/CV2, anti-Hu, anti-Ri, anti-Yo, and anti-GAD65] [65]. For patients with paraneoplastic autoimmune chorea 3 of 7 had improvement with oncologic treatment, and 5 of 11 improved with immunotherapy consisting of corticosteroids alone, plasmapheresis, cyclophosphamide, or corticosteroids with IVIg. Additional chronic non-steroidal treatments included hydroxychloroquine, azathioprine, cyclophosphamide, and mycophenolate mofetil. Based on this study, they concluded that the various antibodies produced by cancers may have multiple cross-reactive neurologic targets within the BG causing over-activation of the direct or inhibition of the indirect pathway. This hypothesis is supported by the improvement of chorea with oncologic treatment and/or immunosuppression.

In general, chorea due to autoimmune or paraneoplastic conditions is likely caused by cross-reactivity of antibodies to elements of the BG resulting in imbalance of the direct/indirect pathways, and abnormally increased thalamic output. Chorea due to autoimmune pathology improve with immunosuppression, IVIg, or plasmapheresis, and chorea due to paraneoplastic diseases improves with treating the underlying cancer and/or immunosuppression.

### Non-HD genetic disorders with chorea

Genetic abnormalities are the most common cause of adult-onset chorea, with HD being the most prevalent genetic disease associated with chorea. Due to the focus of this paper, treatments of HD chorea will not be discussed because it is a primary chorea, in such chorea is central to the pathology of HD rather and secondary. Non-HD genetic diseases characterized by chorea include *C9ORF72* mutation-related neurodegeneration, spinocerebellar ataxia syndromes (SCA’s) including dento-rubral pallidoluysian atrophy, HD-like 1, HD-like 2, benign hereditary chorea syndromes, chorea-acanthocytosis, McLeod syndrome, multiple autosomal recessive ataxia syndromes, neurodegeneration with brain iron accumulation disorders (NBIA), Wilson’s disease, X-linked dystonia parkinsonism (XDP), Lesh-Nyhan syndrome (LNS), paroxysmal kinesigenic dyskinesia (PKD), non-kinesigenic dyskinesias (PnKD), and glucose transporter 1 (GLUT1) deficiency [2,62]. Currently, these genetic syndromes and their associated chorea are treated symptomatically.

Even though chorea may be present in a variety of genetic diseases, there is a dearth of available literature regarding treatment of genetic chorea [75]. This is at least in part due to the fact that many of these disorders are rare, progressive, neurodegenerative disorders, and the performance of double-blind RTCs is challenging in this population. Most treatment is based upon individualized pharmacotherapy and extrapolated from experience in HD.

While there is a paucity of disease-specific treatments for most genetic causes of chorea, some disorders do have unique metabolic targets that may be amenable to intervention. The few reported successful treatments of non-HD genetic chorea are discussed below.

NBIA syndromes are a group of neurodegenerative diseases which are characterized by accumulation of iron in the BG, and present with a range of movement disorders, in addition to cognitive impairment [76,77]. These disorders include aceruloplasminemia (aCP), neuroferritinopathy (Nf), pantothenate kinase-associated neurodegeneration (PKAN), and phospholipase-associated neurodegeneration (PLAN) [77,78,79]. These genetic abnormalities result in deposition of iron in the striatum, thalamus, globus pallidus, dentate nuclei, cortex, retina, liver, and pancreas; causing symptomatic chorea, blepharospasm, dystonia, parkinsonism, oral-lingual-mandibular dystonia, ataxia, and cognitive decline. The significance of the common feature of BG iron deposition in NBIA is not yet fully understood, nor the extent to which removal of iron by chelation is therapeutic, although it is reported to be beneficial in some cases.

aCP is a very rare disease with a prevalence of approximately 1 per 2,000,000; aCP is caused by homozygous mutations of the *CP* gene, resulting in dysfunctional or nonfunctional ceruloplasmin and thus brain iron deposition [80,81,82]. Ceruloplasmin is a multi-copper oxidase protein that oxidizes ferrous iron after it transfers it to extracellular transferrin.

Nf is even rarer with 90 reported cases as of 2016 [83]. Mutations of the *FTL* gene result in intracytoplasmic and intra-nuclear aggregations of ferritin in glial cells resulting in deposition of iron, gliosis, and neuronal death due to abnormal iron storage and oxidation [77,83,84].

The *PANK2* gene codes for pantothenate kinase 2 protein, which regulates the biosynthesis of coenzyme A in mitochondria, and controls the accumulation of N-pantothenoyl-cysteine and pantetheine. A mutation in *PANK2* causes unregulated accumulation of N-pantothenoyl-cysteine and pantetheine, with the end result of iron accumulation in the BG, referred to as PKAN [85].

Due to the rarity of the NBIA syndromes, most of the treatment recommendations are based on few case studies and expert opinion. For patients with aCP, chelating agents decrease serum iron concentrations and brain and liver iron stores with improved BG signal intensity on T2-MRI and may provide some clinical benefit [86]. Desferrioxamine 1000 mg i.v./d for 5 days followed by deferiprone 500 mg/day in addition to 500 mg iv fresh frozen plasma with ceruloplasmin resulted in at least 6 months of improved gait, trunk ataxia, and myoclonus (without mention of chorea) for a patient with aCP [86]. Skidmore et al. reported a 54-year-old woman with aCP who had improvement in chorea and ataxia after three months of Deferasirox 1000 mg/day [87].

Similar to aCP, deferiprone 15mg/kg twice daily potentially improves ataxia, gait, and chorea for patients with NBIA [76,78]. The initial case report of a 61 year old woman with NBIA treated with hydroxypyridone deferiprone 15 mg/kg twice a day had improvement in chorea and gait with reduction of iron depositions in BG on MRI with 6 months of treatment [78]. This case inspired a small multi-center nonblinded trial of deferiprone 15 mg/kg twice a day for safety and efficacy, showed mild to moderate motoric improvement in 3 of 11 NBIA patients, 2 of whom had PKAN [76]. These rare cases of NBIA with chorea improving with chelation demonstrates the need for larger, double-blinded treatment studies to provide more options for treating these rare diseases.

In contrast to aCP, for patients with Nf chelation is not clinically beneficial; there are reports of chorea improvement with DA modulating agents [88,89,90]. Sulpiride 400 mg/day and TBZ up to 125 mg/day both improved chorea, whereas desferrioxamine 4000 mg weekly for 14 months and deferiprone 15mg/kg/day for 6 months were not effective at treating motor features of Nf [88,90].

Some non-HD chorea genetic disorders improve with dietary changes and do not require aggressive and potentially harmful medical interventions. GLUT1 deficiency is a genetic disorder of impaired glucose transportation across the blood-brain barrier due to mutations in the *SLC2A1* gene. This can result in an array of neurologic symptoms including paroxysmal exercise-induced chorea, dystonia, and epilepsy. Symptoms may improve with strict adherence to the ketogenic diet [91].

PDK is caused by mutations in the gene encoding for proline-rich transmembrane protein 2 (PRRT2), presenting clinically with brief sudden onset unilateral or bilateral attacks of dystonia and chorea that is brought on by sudden movements [92,93]. Most patients respond to phenytoin and CBZ, alternative therapies include benzodiazepines and other AEDs such as VPA, gabapentin, lamotrigine, levetiracetam, and oxcarbazepine [94,95]. PnKD is commonly caused by mutations in the myofibrillogenesis factor 1 gene (*MR1*), resulting in episodic bouts of involuntary moments such as chorea, dystonia, and ataxia that are triggered by caffeine, fatigue, alcohol, stress, intense emotions, and laughter. Benzodiazepines and trigger avoidance are the most effective treatments for preventing all PnDK symptoms including chorea [96]. Chorea secondary to GLUTI and PnDK can be controlled with strict dietary restrictions, AEDs, and trigger avoidance.

Wilson’s disease is caused by a mutation of the *ATP7B* gene resulting in decreased biliary copper excretion, hence copper accumulates in the brain resulting in multiple neurologic symptoms including parkinsonism, chorea, and dystonia. Chelation with trientine, D-penicillamine, tetrathiomolybdate, and zinc effectively reduce copper deposition and result in the improvement of the movement disorders associated with Wilson’s disease [97].

For many of these genetic diseases chorea is a minor symptom, and there are other symptoms such as psychiatric, dysphagia, dysarthria, and gait disorders that have a much larger impact on the patient’s and the caregiver’s quality of life. An integrated multidisciplinary approach involving neurology, psychiatry, physical and occupational therapy, case management, nursing, and strong social support structures is the ideal management for such patients.

## Conclusion

When initiating treatment for secondary chorea, the medical provider must determine the underlying medical abnormality and begin appropriate therapy. In this review we critically analyzed the available literature on the treatment of secondary chorea by assessing available expert clinical experience, case reports, case series, open-label trials, and RTC in order to provide secondary chorea treatment recommendations (Table 1). Given the limited number of RTCs, there is a significant lack of EBM regarding chorea treatment and most available recommendations are derived from expert clinical experience, case reports, or small open-label studies. These limitations are in part due to the self-resolving nature of most chorea; and for non-HD genetic causes of chorea, research is limited due to rarity of these diseases and their varied progression rates. TD treatment has the most robust scientific evidence supporting the use of VMAT2 inhibitors, which is made possible by employing consistent use of standardized formal chorea scoring and disease ubiquity. Regardless of the cause of secondary chorea, there are very few placebo-controlled trials guiding treatments; hence formal guidelines cannot be formulated based on the current literature and future RTCs would provide evidence-based data for treatments.

Chorea due to infectious causes is likely due to molecular mimicry where disease-specific antibodies cross-react with dopaminergic neurons in the BG resulting in an imbalance of the direct and indirect pathways. Treatment of the underlying infection decreases the amount of circulating antibodies and normalizes BG function.

TD has been studied much more rigorously than the other conditions described here. The development of VBZ and potential for more selective VMAT2 inhibitors may further improve treatment for TD.

Chorea has been reported in the setting of multiple different types of metabolic abnormalities, including hyperthyroidism, vitamin B12 deficiency, and pregnancy. The underlying cause of chorea in the setting of metabolic derangement is unclear, however it typically resolves as the metabolic abnormality improves. Only case studies and case reports are available on this topic. Increasing this literature to include case series would be beneficial to guiding practitioners.

Autoimmune and paraneoplastic associated chorea is likely due to autoantibodies crossing the blood brain barrier and binding to the BG causing upset in normal function. Treatments include antiplatelet agents, anticoagulation, and immunosuppression based on expert clinical experience and few case series.

For patients with non-HD genetic chorea, treatment is based on expert clinical practice and case reports. Given the rarity of these genetic diseases and the variable rates at which they progress, RTCs are nearly impossible to complete. For these rare diseases expert opinion provides the best possible guidelines for treatments.

This review highlights the marked lack of EBM in the treatment of secondary chorea, and the barriers to double-blind, randomized controlled studies. Given the limited EBM in this filed, expert opinion and the clinical experiences of practiced physicians are fundamental in guiding chorea management and determining successful treatment. The quality of chorea research would improve with standard implementation of formal chorea scoring techniques including direct observation by experienced practitioners and the use of accelerometer and other motion detecting devices to provide objective chorea scoring. In conclusion, there is a strong need for well-controlled studies to develop clear guidelines for the most beneficial treatments of secondary chorea.
